# The double orifice valve technique to treat tricuspid valve incompetence

**DOI:** 10.1186/1749-8090-8-S1-O103

**Published:** 2013-09-11

**Authors:** R Hetzer

**Affiliations:** 1 Cardiothoracic Surgery, Deutsches Herzzentrum Berlin, Berlin, Germany

## Objectives

This aims to illustrate and report a straightforward tricuspid valve (TV) repair technique employed for moderate or severe functional (normal valve with dilated annulus) or for primary /organic (Ebstein’s anomaly, leaflet or chordal retraction/tethering, with annular dilatation) tricuspid valve incompetence and its long-term outcome.

## Methods

The double-orifice valve technique was employed on 73 patients (mean age 52.6±23.2 years) with severe tricuspid regurgitation: 3 post-transplant iatrogenic chordal rupture, 23 Ebstein's anomaly, and 47 solated severe TV incompetence. The basic principle is to reduce the distance between the coapting leaflets by creating two orifices. The degree and extent of creating a double valve orifice was determined by considering the minimal body surface area (BSA)-related acceptable TV diameter. Repair was accomplished by ledgeted mattress sutures from the middle of the true anterior annulus to a spot on the opposite septal annulus avoiding injury to the Bundle of His.The annular apposition divides the TV into having a larger anterior and a smaller posterior orifices, enabling valve closure. In adults, the diameter of the anterior valve orifice should be 23 to 25 mm, and the posterior orifice should be 15-18 mm.

## Results

At a mean follow-up of 4.1 years (range 1.5-12.4 years), there has been no reoperation for tricuspid valve insufficiency or stenosis. Reoperations done on 3 patients were indicated in one for aortic valve replacement 14 months postoperatively and in 2 for assist device implantation who eventually underwent heart transplantation 18 and 20 months aftert TV repair, respectively. Cumulative 12-year survival rate is 88%.

## Conclusions

This technique is technically a straightforward repair to abolish TV incompetence with highly satisfactory results. It provides no pitfalls, if one keeps in mind the location of the conduction system, and neither residual regurgitation nor reoperation occurs.

